# Health-Related Quality of Life and Related Characteristics of Informal Caregivers Providing Home Health Care to Elderly Patients: A Cross-Sectional Study

**DOI:** 10.3390/healthcare14081084

**Published:** 2026-04-18

**Authors:** Yusuf Adnan Güçlü, Nil Tekin, Şerafettin Ceylan

**Affiliations:** 1 Department of Family Medicine, Tepecik Research and Trainning Hospital, Izmir Faculty of Medicine, Health Sciences University, Izmir 9035460, Türkiye; niltekin33@yahoo.com; 2 Alsancak State Hospital, Health Ministerium, Izmir 9035220, Türkiye; serefceylan@gmail.com

**Keywords:** informal caregiver, health-related quality of life, SF-36, home health care, palliative performance scale

## Abstract

**Highlights:**

**What are the main findings?**

- Caregiver quality of life subdimensions are associated with sociodemographic factors and the patient’s level of palliative care needs.
- Comparing caregiver QoL scores with national norms identifies the most affected health dimensions, highlighting priority areas for intervention.

**What are the implications of the main findings?**

- The results of this study suggest that in an aging population, the health of caregivers is at significant risk in all dimensions compared with the general population. The findings highlight the profound impact of caregiving on family health, which can be conceptualized as affecting not only the patient but also the well-being of informal caregivers.
- Health systems urgently need to implement policies and practices that recognize, support, and empower caregivers, moving beyond treating them as “invisible patients.”

**Abstract:**

**Objective:** This study aimed to evaluate the health-related quality of life (HRQoL) of informal caregivers providing primary care to elderly chronically ill patients receiving home health care services in Türkiye and to identify patient and caregiver characteristics independently associated with HRQoL. **Methods:** This cross-sectional study included 499 patient–caregiver dyads enrolled in home health care services at a training and research hospital in İzmir, Türkiye. Data were collected through face-to-face interviews using a sociodemographic questionnaire, the Palliative Performance Scale (PPS), and the Short Form 36 (SF-36). One-sample *t*-tests compared SF-36 scores with Turkish normative values. Multivariate linear regression identified independent predictors of the Physical Component Summary (PCS) and Mental Component Summary (MCS) scores. **Results:** Caregivers scored significantly lower than population norms across all SF-36 subdimensions (*p* < 0.001), with the largest impairments in Role Physical (mean difference: −53.0) and Role Emotional (−42.9). In multivariate analyses, independent predictors of poorer physical health (PCS) were severe patient functional dependence (PPS ≤ 30: β = −0.260, *p* < 0.001), older caregiver age (≥65 years: β = −0.089, *p* = 0.044), unemployment (β = −0.118, *p* = 0.014), additional care recipients (β = −0.095, *p* = 0.026), and caregiver’s own chronic illness (β = −0.169, *p* < 0.001). Poorer mental health (MCS) was independently associated with caregiver’s own chronic illness (β = −0.138, *p* = 0.002), receipt of caregiving payment (β = −0.137, *p* = 0.004), and university-level education (β = −0.108, *p* = 0.040), whereas the presence of a support person was protective (β = 0.096, *p* = 0.038). **Conclusions:** Informal caregivers of home health care-dependent elderly patients experience significantly reduced quality of life across all health domains compared with the general population. The independent determinants of caregiver health are multidimensional, encompassing patient-related factors, socioeconomic characteristics, and psychosocial resources. These findings underscore the urgent need for health systems to implement tailored interventions that address the distinct physical and mental health needs of caregivers, with particular attention to those who are elderly, chronically ill, socioeconomically disadvantaged, or highly educated.

## 1. Introduction

Globally, increased life expectancy and demographic shifts have led to a dramatic rise in the prevalence of chronic non-communicable diseases and associated chronic disabilities [1]. This epidemiological shift has moved care services from hospital-centered models to community- and home-based approaches, making informal caregivers the “invisible backbone” of the health system [2]. Family members, often without professional training, make significant sacrifices to their own lives and health while meeting patients’ physical, emotional, and administrative needs [3,4]. Providing care triggers “caregiver burden,” a multidimensional stressor resulting from an imbalance between an individual’s capacity and the demands of care [5]. The literature consistently documents that this burden is directly linked to serious psychosocial morbidities, including high levels of anxiety, depression, sleep disorders, and social isolation [6,7]. Informal caregivers become “hidden patients” of the system, neglecting their own health while managing the patient’s symptoms and uncertain prognosis, which can negatively affect the quality of care provided [8,9].

General health screening tools, such as the SF-36, show that caregivers experience significant declines, particularly in physical role functioning, vitality, and mental health, compared with general population norms [10]. This multidimensional assessment of physical, psychological, and social functioning is considered a critical outcome measure in caregiver research [4,11]. Factors such as the patient’s advanced age and level of functional dependence, as well as the caregiver’s gender and low income, have been shown to play a significant role in this decline [12,13]. Furthermore, the dynamic interaction between patient and caregiver creates a system of interdependence, in which deterioration in one party’s health or psychological stress directly affects the quality of life and well-being of the other [14,15]. Caring for advanced-stage patients in need of care is among the most stressful processes due to the severity of symptoms and the reality of impending death [5,7].

In countries such as Türkiye, where traditional family ties are strong, but the population is rapidly aging, the central role of family care is under increasing pressure. The current literature in Türkiye generally focuses on specific disease groups [16,17]; however, comprehensive studies are needed that compare the quality of life (QoL) of caregivers caring for a heterogeneous patient group with low palliative performance levels with national population norms, while also analyzing the dyadic factors influencing this relationship. In this study, we aim to assess the QoL of primary family caregivers of home care patients in İzmir, Türkiye, using the SF-36 and to determine the magnitude of the difference relative to population norms. A secondary objective is to identify the most at-risk groups by examining how patient and caregiver characteristics affect different domains of QoL. The findings are expected to provide an evidence-based framework for developing clinical interventions and public health policies to support caregivers.

## 2. Materials and Methods

### 2.1. Study Design and Setting

This cross-sectional, descriptive, and analytical study was designed to examine the quality of life of caregivers in chronic disease management and home health care, as well as the determinants of caregiver quality of life. The study was conducted in full compliance with the STROBE (Strengthening the Reporting of Observational Studies in Epidemiology) guidelines, which were developed to improve the reporting quality of observational studies. The study population consisted of patients receiving home health care services at the Health Sciences University, Izmir Tepecik Training and Research Hospital, and their primary caregivers between November and December 2025.

### 2.2. Participants

Participants were recruited using the patient–caregiver dyad approach [17,18]. All patients enrolled in the hospital’s home health care services and their primary informal caregivers who met the inclusion criteria were approached consecutively during routine care team visits or by phone and invited to participate. A total of 499 dyads who agreed to participate and met the criteria were included. For patients, inclusion criteria were as follows: (1) age ≥ 65 years; (2) receiving home health care; and (3) having at least one diagnosed chronic disease. For caregivers, inclusion criteria were as follows: (1) age ≥ 18 years, (2) actively undertaking primary care responsibilities for at least three months, and (3) having the cognitive capacity to understand the study [19]. End-of-life patients and caregivers with known serious psychiatric diagnoses were excluded from the study to maintain data validity [20]. Cognitive capacity was assessed clinically by the attending physician based on routine clinical evaluation, and patients with obvious cognitive impairment (e.g., advanced dementia) were excluded. Psychiatric diagnoses were verified through patient medical records. During the November–December 2025 recruitment period, 560 eligible dyads were approached; of these, 61 (10.9%) declined to participate, primarily citing lack of time or unwillingness to be interviewed. The final sample thus consisted of 499 dyads (response rate: 89.1%).

### 2.3. Measures and Data Collection

The sample size was calculated *a priori* using G*Power (version 3.1.9.7) to ensure adequate statistical power. Based on 80% power, a 5% significance level, and an effect size of 0.15 (Cohen’s f) [21], a minimum of 450 dyads were required to detect a 5-point clinical difference in SF-36 subdimensions. Accounting for a potential 10% data loss, a total of 499 patient–caregiver dyads were included in the analysis.

Data were collected through face-to-face interviews with the caregivers conducted by a team of three researchers (medical doctors). Prior to data collection, all interviewers completed a standardized training session led by the principal investigator to ensure consistency in questionnaire administration, including the use of uniform prompts and clarification of potential ambiguities. Regular meetings were held throughout the data collection period to maintain standardization. The data were collected using a sociodemographic questionnaire for caregivers and patients, the Palliative Performance Scale (PPS), which measures the patient’s functional status, and the SF-36, which assesses health-related quality of life.

1. Sociodemographic Questionnaire: This questionnaire captured sociodemographic and caregiving-related variables (age, sex, education level, income level, marital status, employment status, presence of chronic illness) for both caregivers and patients. Variable selection was guided by a comprehensive literature review, which consistently identifies these factors as significant determinants of caregiver burden and health-related quality of life [12,13,19]. For caregivers, we also collected data on caregiving duration, receipt of caregiving payment, having additional care recipients, and availability of a support person.
2. Palliative Performance Scale (PPS): The PPS was used to objectively assess the patient’s functional status and performance level [22]. The PPS is a valid and reliable tool that evaluates five basic criteria “mobility, activity and self-care, food/fluid intake, and level of consciousness” yielding a total score ranging from 10% (terminal stage, near death) to 100% (complete independence) [23,24]. Lower scores reflect greater physical dependence and the need for palliative care. In this study, PPS scores were categorized into commonly accepted groups to facilitate clinical application and statistical analyses: Mildly Dependent (PPS ≥ 70), Moderately Dependent (PPS 40–60), and Severely Dependent (PPS ≤ 30) [25].
3. SF-36 Health Questionnaire: Caregivers’ self-report (SR) was assessed using the 36-item Short Form Health Questionnaire (SF-36), a widely used, multidimensional, general self-report scale [26]. The scale comprises eight subdimensions: physical functioning (PF), physical role difficulty (RP), bodily pain (BP), general health perception (GH), vitality (VT), social functioning (SF), emotional role difficulty (RE), and mental health (MH). Raw scores for each dimension were converted to a 0–100 scale, with 0 representing the worst health status and 100 the best. The validity and reliability of the Turkish version of the SF-36 were established by Koçyiğit et al. [27]. Normative data established for the Turkish population were used as reference values to compare SR scores with those of the general population [28]. These normative data served as the basis for quantifying the magnitude (effect size) of deviations in the SR population across the SF-36 subdimensions. Physical Component Summary (PCS) and Mental Component Summary (MCS) scores were calculated as the mean of the respective subscales. The PCS was derived as the average of Physical Functioning (PF), Role Physical (RP), Bodily Pain (BP), and General Health (GH) domains. The MCS was derived as the average of Vitality (VT), Social Functioning (SF), Role Emotional (RE), and Mental Health (MH) domains. Higher scores indicate better health-related quality of life.

### 2.4. Statistical Analysis

Statistical analyses were performed using IBM SPSS Statistics 25 and R (version 4.2.1). Descriptive statistics were reported as counts (n) and percentages (%) for categorical variables, and as means ± standard deviations (SD) for continuous variables. There were no missing data for the primary variables. Internal consistency reliability of the study instruments was assessed using Cronbach’s alpha coefficient. In this study, Cronbach’s alpha values for the SF-36 subscales were as follows: Physical Functioning (PF) 0.85, Role Physical (RP) 0.88, Bodily Pain (BP) 0.79, General Health (GH) 0.76, Vitality (VT) 0.81, Social Functioning (SF) 0.74, Role Emotional (RE) 0.89, and Mental Health (MH) 0.82. The Palliative Performance Scale (PPS) demonstrated a Cronbach’s alpha of 0.71. All values exceeded the commonly accepted threshold of 0.70, indicating acceptable to good internal consistency for the instruments used in this study sample.

To evaluate differences between caregiver SF-36 domain scores and Turkish population norms [28], one-sample *t*-tests were performed. To quantify the magnitude of these differences, effect sizes were calculated using Hedges’ g and are reported with 95% confidence intervals (CIs). Hedges’ g was chosen as it provides a conservative estimate of effect size and is recommended for precise reporting, thereby facilitating future meta-analyses [29]. Effect sizes were interpreted according to Cohen’s criteria: small (0.2), medium (0.5), and large (0.8).

Normality of the SF-36 domain scores was assessed using the Shapiro–Wilk test and visual inspection of Q-Q plots. While most domains approximated normality, the Role Physical (RP) domain exhibited significant deviations from normality (Shapiro–Wilk *p* < 0.001), consistent with its known distributional properties due to ceiling and floor effects. Therefore, for bivariate comparisons involving the RP domain, non-parametric tests were employed: the Mann–Whitney U test for two-group comparisons and the Kruskal–Wallis test for comparisons across three or more groups. For all other SF-36 domains, parametric tests (independent-samples *t*-test, one-way ANOVA) were applied. To maintain consistency and interpretability, mean ± standard deviation values are reported for all domains in the descriptive tables, as is common practice in health-related quality of life research.

To identify factors independently associated with caregiver quality of life, we subsequently performed multivariate linear regression analyses. Two separate models were constructed with the Physical Component Summary (PCS) and Mental Component Summary (MCS) scores as dependent variables. These summary scores were calculated based on the standard SF-36 scoring algorithm. The independent variables entered into the models were patient’s PPS level, caregiver’s age, income, education, employment status, presence of chronic illness, availability of a support person, and caregiving duration. The enter method was used. Results are presented as unstandardized coefficients (B) with 95% CIs and standardized coefficients (β). Multicollinearity was assessed using Variance Inflation Factor (VIF). Statistical significance was set at *p* < 0.05.

## 3. Results

This study included 499 patient–caregiver dyads. The sociodemographic, clinical, and caregiver characteristics of the participants are presented in Table 1. The mean age of the patients was 80.4 (±7.2) years, and 61.5% were female. Notably, 34.9% of patients were aged 85 years or older. Regarding caregiver characteristics, the vast majority (85.0%) were aged 18–64 years, and 72.1% were female. In addition, 75.2% of caregivers were married, 76.2% were in the middle-income group, and 76.0% had a high school education or less. Moreover, 51.5% of caregivers reported having a chronic disease, and 60.7% reported having an additional support person. Regarding the chronic disease status of the caregivers, the most prevalent conditions among those with at least one morbidity (n = 257) were hypertension (52.3%), diabetes mellitus (29.7%), and other conditions (38.3%), which included a range of musculoskeletal, neurological, and gastrointestinal disorders. Regarding the chronic disease status of the patients, the most common conditions were hypertension, heart diseases, diabetes, dementia, cancer and cerebrovascular diseases. As indicated in Table 1, a significant proportion of these patients, 146 (29.3%), had four or more concomitant diseases, highlighting the high level of clinical complexity.

Table 2 presents a comparison of caregiver SF-36 quality-of-life scores with Turkish population norms. Caregivers’ scores across all SF-36 subdimensions were significantly lower than normative values (*p* < 0.001 for all). The largest differences were observed in the Role Physical (RP: −53.0, Hedges’ g = −1.347) and Role Emotional (RE: −42.9, Hedges’ g = −1.442) subdimensions. Effect sizes (Hedges’ g) ranged from −0.359 (Physical Function) to −1.442 (Role Emotional), indicating large or moderate-to-large effects.

Table 3 presents the distribution of SF-36 scores according to patient and caregiver characteristics. When analyzing patient-related factors, a significant difference was observed only in the Mental Health (MH) domain across patient age groups (*p* = 0.027). Post hoc Tukey HSD test revealed that caregivers of patients aged 65–74 years had significantly lower MH scores (48.9 ± 15.1) compared with those caring for patients aged 75–84 years (53.5 ± 16.1, *p* = 0.024). No significant differences were found in other domains (PF: *p* = 0.093; RP: *p* = 0.087; BP: *p* = 0.281; GH: *p* = 0.662; VT: *p* = 0.285; SF: *p* = 0.103; RE: *p* = 0.755). Regarding patient sex, caregivers of female patients had significantly higher General Health (GH) scores (58.5 ± 18.7 vs. 54.6 ± 18.4, *p* = 0.024), while Bodily Pain (BP) showed a borderline significant difference (*p* = 0.059). No significant differences were observed across patient comorbidity groups in any SF-36 domain (all *p* > 0.05). However, significant differences were found across patient Palliative Performance Scale (PPS) groups in Physical Functioning (PF: *p* = 0.009), Role Physical (RP: *p* < 0.001), and Bodily Pain (BP: *p* < 0.001). Post hoc analyses indicated that caregivers of mildly dependent patients (PPS ≥ 70) had significantly higher RP and BP scores than those caring for moderately or severely dependent patients.

Regarding caregiver characteristics, caregivers aged 18–64 years had significantly higher scores in PF (*p* < 0.001), GH (*p* = 0.003), VT (*p* = 0.008), SF (*p* = 0.002), and MH (*p* = 0.016) compared with those aged ≥ 65 years. Male caregivers scored significantly higher in PF (*p* = 0.034), RP (*p* = 0.036), and BP (*p* < 0.001) than female caregivers. Caregivers with higher education (university or above) had significantly higher PF and RP scores (both *p* < 0.001) but significantly lower SF (*p* = 0.033) and MH (*p* < 0.001) scores compared with those with high school education or less. Employed caregivers had significantly higher scores in PF, RP, BP, GH, and VT (all *p* < 0.01) than unemployed caregivers.

Caregivers with additional care recipients had significantly lower PF (*p* = 0.016) and RP (*p* = 0.012) scores than those without. Caregivers with chronic illness had significantly lower scores across all subdomains except RE and MH (PF, BP, GH, VT, SF: all *p* < 0.01). The presence of a support person was associated with significantly higher PF (*p* < 0.001), GH (*p* = 0.031), and SF (*p* = 0.002) scores. Regarding caregiving duration, significant differences were observed in PF (*p* < 0.001), with post hoc analysis showing that caregivers with 0–1 years of caregiving had significantly higher PF scores than ≥6 years (*p* < 0.001), and those with 2–5 years had higher PF scores than those with ≥6 years (*p* = 0.017).

To provide a summary assessment of caregivers’ overall physical and mental health, we computed the Physical Component Summary (PCS) and Mental Component Summary (MCS) scores from the SF-36 subscales. The distribution of these summary scores according to patient and caregiver characteristics is presented in Table 4.

In bivariate analyses, lower PCS scores were significantly associated with greater patient functional dependence (PPS ≤ 60), older caregiver age (≥65 years), male caregiver sex, lower income, lower education level, unemployment, presence of additional care recipients, caregiver’s own chronic illness, absence of a support person, and longer caregiving duration (all *p* < 0.05). Lower MCS scores were significantly associated with older caregiver age (≥65 years), higher education level (university or above), receipt of caregiving payment, caregiver’s own chronic illness, and absence of a support person (all *p* < 0.05). Detailed post hoc comparisons are provided in the table notes.

To identify factors independently associated with caregiver quality of life while accounting for potential confounders, we performed multivariate linear regression analyses with the Physical Component Summary (PCS) and Mental Component Summary (MCS) scores as dependent variables. The results are presented in Table 5. In the PCS model, significant independent predictors of lower physical health were moderate patient dependency (PPS 40–60: B = −8.56, 95% CI [−13.14, −3.98], *p* < 0.001), severe patient dependency (PPS ≤ 30: B = −12.05, 95% CI [−17.44, −6.65], *p* < 0.001), caregiver age ≥ 65 years (B = −4.72, 95% CI [−9.32, −0.12], *p* = 0.044), unemployment (B = −4.98, 95% CI [−8.95, −1.01], *p* = 0.014), presence of additional care recipients (B = −4.10, 95% CI [−7.71, −0.49], *p* = 0.026), and caregiver’s own chronic illness (B = −6.40, 95% CI [−9.58, −3.22], *p* < 0.001). The model explained 16.9% of the variance in PCS scores (R^2^ = 0.169, adjusted R^2^ = 0.145, *p* < 0.001). In the MCS model, significant independent predictors of lower mental health were caregiver’s own chronic illness (B = −4.71, 95% CI [−7.75, −1.67], *p* = 0.002), receipt of caregiving payment (B = −5.76, 95% CI [−9.71, −1.81], *p* = 0.004), and university-level education (B = −4.31, 95% CI [−8.42, −0.20], *p* = 0.040). The presence of a support person was independently associated with better mental health (B = 3.37, 95% CI [0.19, 6.55], *p* = 0.038). The MCS model explained 7.1% of the variance (R^2^ = 0.071, adjusted R^2^ = 0.044, *p* = 0.001).

## 4. Discussion

This study provides a detailed analysis of the health-related quality of life of informal caregivers of elderly, chronically ill patients requiring home health care in a major urban center in Türkiye (İzmir), comparing their QoL with national population norms and examining factors related to both patient and caregiver characteristics. Our findings confirm that the caregiving process profoundly affects caregivers’ health not only in specific domains but across the biopsychosocial spectrum. The profound deviations observed, particularly in physical and emotional role difficulties, clearly reveal that this population has become the “hidden patients” of the health system [9,30].

The most striking result of the study is that the informal caregivers’ scores on all eight SF-36 subdimensions are both statistically and clinically lower than the general population norms in Türkiye. The greatest impairments were observed in the Role Physical (RP) and Role Emotional (RE) dimensions, indicating that caregivers’ capacity to maintain daily activities and social roles was severely affected. This finding aligns with the thesis in the literature that “caregiver burden” is not only a psychological stressor but also a multidimensional phenomenon that triggers physical morbidity and paralyzes the individual’s social functioning [31,32].

Our findings demonstrate a direct correlation between the patient’s level of functional dependence and the caregiver’s overall health. As the PPS score decreases, particularly with worsening physical function and bodily pain, the intensive physical support required by advanced-stage patients depletes caregivers’ musculoskeletal health and energy reserves. Sources in the literature consistently highlight that as the patient’s symptom severity and degree of dependence increase, the caregiver’s “objective burden” increases, directly impacting health outcomes [19,31].

In our study, caregivers aged 65 years and older reported significantly lower overall health than their younger counterparts. This group of “elderly caregivers” faces chronic diseases and declining physiological reserves related to their own aging process, while simultaneously undertaking heavy caregiving responsibilities [1,9]. These findings underscore the need for health systems to identify this subgroup as a priority risk group and to develop customized support models for them [1,33].

The finding that low income negatively affects all areas of caregiving confirms that financial constraints are a “secondary stressor” that deepens stress in the caregiving process [33]. However, the “education paradox” identified in our study, in which highly educated caregivers have high physical scores but low mental health scores, is critical. Highly educated individuals, with greater access to information about the disease process and a stronger sense of “locus of control,” may experience exacerbated feelings of uncertainty and helplessness [34,35]. Even individuals with high intrapersonal emotional competence may be overwhelmed by the interpersonal burden of caregiving [35]. This situation can be conceptualized as the “psychological burden of information.” Furthermore, the conflict between the social and professional identities of educated caregivers and their caregiving role may be deeper. This finding indicates that caregiver support programs should be “education-level sensitive”. However, this hypothesis requires further validation through qualitative or mixed-methods research.

The multivariate analysis extended the bivariate findings by revealing distinct patterns of association for physical and mental health outcomes, highlighting the multidimensional nature of caregiver burden. The persistent and dose-dependent effect of patient functional dependence on caregivers’ physical health with a nearly twofold stronger effect for severe versus moderate dependency suggests that the physical demands of caregiving constitute a primary pathway through which patient illness directly compromises caregiver well-being. This finding aligns with the “wear and tear” hypothesis, which posits that cumulative physical strain from caregiving tasks accelerates physiological decline [19,31]. The absence of a similar effect on mental health in the adjusted model further supports this interpretation, indicating that the psychological impact of patient dependency may be largely mediated by other factors such as perceived burden or coping resources rather than by functional status itself.

Among caregiver characteristics, the independent association of chronic illness with both physical and mental health outcomes underscores the vulnerability of caregivers who themselves face health limitations, a phenomenon previously described as the “hidden patient” paradox [9]. Notably, while unemployment and additional caregiving responsibilities negatively affected physical health, their effects on mental health were attenuated after adjustment, suggesting that the psychological toll of these stressors may be partially explained by financial strain or social isolation rather than their direct effects. Conversely, the persistent negative association between university education and mental health despite its positive association with physical health robustly supports the “education paradox” hypothesis. This counterintuitive finding may reflect heightened awareness of disease prognosis, increased perceived responsibility, or internalized pressure to meet idealized caregiving standards among more educated individuals [34,35]. Such individuals may also experience greater role conflict between professional identity and caregiving demands, leading to a “psychological burden of information” that is not captured by physical health measures.

The divergent patterns observed between the PCS and MCS models underscore the multidimensional nature of caregiver burden. While patient-related factors (functional dependence) predominantly influenced physical health, caregiver-related psychosocial factors (chronic illness, financial strain, education) were more salient for mental health. This dissociation suggests that interventions aimed at improving caregiver well-being must be tailored to address the specific determinants of physical versus mental health outcomes.

The protective effect of a support person on mental health, independent of other factors, reinforces the critical role of social networks in mitigating caregiver distress. This aligns with the buffering hypothesis of social support, which posits that supportive relationships provide emotional resources and practical assistance that reduce the impact of stressors [5]. However, the lack of a significant protective effect on physical health suggests that while social support may alleviate psychological strain, it may not sufficiently offset the tangible physical demands of caregiving. Collectively, these findings suggest that interventions targeting caregiver well-being must be tailored to address the distinct determinants of physical and mental health outcomes, with a particular focus on providing practical support for physically demanding tasks, psychosocial support for highly educated caregivers, and strengthening social networks to enhance psychological resilience.

The presence of a chronic illness among informal caregivers was associated with the greatest decline in QoL scores; this finding supports dyadic interaction models, which emphasize that the patient–caregiver dyad should be considered as a whole [15,33,36]. In light of our findings, traditional patient-focused models should be replaced with integrated approaches that regularly monitor caregiver burden (Zarit, SF-36, etc.) and include psychoeducational support programs such as the COPE model [8,15]. In particular, the protective effect of social support networks on caregiver burden underscores the importance of integrating community-based support mechanisms and digital health platforms into clinical practice [5,37]. Specifically, routine screening of caregiver health using brief tools such as the SF-12 or Zarit Burden Interview, integrated within home care visits, could facilitate early identification of at-risk individuals. Furthermore, targeted psychoeducational programs should be developed for highly educated caregivers to address the “psychological burden of information,” while practical respite care services should be prioritized for caregivers with heavy physical demands.

### 4.1. Implications for Research, Practice, and Policy

Longitudinal and qualitative studies are needed to understand why highly educated caregivers, despite being in good physical condition, frequently report poor mental health; accordingly, home care teams should view caregivers as individuals with their own health needs, offering practical assistance such as respite care and home support to those experiencing physical fatigue, while providing personalized psychoeducation to those experiencing emotional difficulties. Furthermore, health systems should formally recognize caregivers as a vulnerable group by mandating routine caregiver health checks during home visits, providing financial support to low-income families, and creating rest services to prevent caregivers from bearing the burden alone.

### 4.2. Limitations

This study has several methodological limitations. First, due to its cross-sectional design, causal relationships among the variables cannot be established. Longitudinal studies are needed to examine the dynamic, time-varying effects of the caregiving process on the caregiver. Second, the data were collected from a single center in one geographic location (İzmir), which limits the generalizability of our findings to other regions or rural areas of Türkiye. Third, the normative values used in the SF-36 comparisons are derived from the general population and have not been specifically standardized to the age-sex distribution of our caregiver group; this factor may have introduced a small degree of bias into the comparisons. Fourth, protective factors such as “support person” have not been qualitatively defined (e.g., type of support, frequency, and degree of closeness). It is recommended that this concept be addressed with more objective criteria (e.g., Multidimensional Scale of Perceived Social Support) in future studies. Fifth, the normative data used for comparison, while the most comprehensive available for Türkiye, are nearly two decades old [28]. Population health perceptions may have shifted over this period, which could introduce a period effect bias. Future research should aim to establish updated national norms. Sixth, the use of self-reported measures may introduce recall and social desirability bias, although standardized interviewer training was implemented to minimize this risk. Seventh, our initial analysis was limited to bivariate comparisons; we addressed this by conducting multivariate regression, which strengthens the validity of our independent associations. However, the possibility of residual confounding from unmeasured variables (e.g., caregiver coping styles, specific social support quality) remains. Lastly, the underlying mechanisms of the “mental health paradox” (good physical functioning but low MH score) observed in highly educated caregivers, such as excessive access to information, existential anxiety, and conflict in perceived control, could not be tested with the quantitative data of this study. Qualitative or mixed-methods designs are needed to validate this hypothesis.

## 5. Conclusions

In conclusion, this single-center study demonstrates that informal caregivers of home health care-dependent elderly patients in İzmir, Türkiye, experience significantly lower health-related quality of life across all SF-36 domains compared with the general population, with the most pronounced impacts on their ability to perform physical and emotional roles. The findings suggest that caregiver QoL is independently associated with a combination of patient-related factors (functional dependence) and caregiver-related factors (age, income, education, chronic illness, and social support). The complex relationship observed between higher education and poorer mental health warrants further exploration. Our findings highlight the critical need for health care systems to recognize caregivers as a vulnerable group. Targeted interventions, particularly psycho-educational support programs and community-based support networks, are urgently needed to safeguard the well-being of these “hidden patients,” especially those who are elderly, have low income, or have chronic diseases themselves.

## Figures and Tables

**Table 1:** Sociodemographic, Clinical, and Caregiving Characteristics of Patient–Caregiver Dyads (N = 499).

Variable	Categories	n (%)
Patient Characteristics		
Age Group	65–74 years	136 (27.3)
	75–84 years	189 (37.9)
	≥85 years	174 (34.9)
Sex	Female	307 (61.5)
	Male	192 (38.5)
Number of Comorbidities	1	69 (13.8)
	2	153 (30.7)
	3	131 (26.3)
	≥4	146 (29.3)
Palliative Performance Scale	Mildly Dependent (≥70)	72 (14.4)
	Moderately Dependent (40–60)	322 (64.5)
	Severely Dependent (≤30)	105 (21.0)
Caregiver Characteristics		
Age Group	18–64 years	424 (85.0)
	≥65 years	75 (15.0)
Sex	Female	360 (72.1)
	Male	139 (27.9)
Marital Status	Married	375 (75.2)
	Not Married	124 (24.8)
Income Level	Low	69 (13.8)
	Middle	380 (76.2)
	High	50 (10.0)
Education Level	High School or Less	379 (76.0)
	University or Higher	120 (24.0)
Employment Status	Employed	139 (27.9)
	Not Employed	360 (72.1)
Caregiving Payment	Receives Payment	105 (21.0)
	No Payment	394 (79.0)
Additional Care Recipient	Yes	129 (25.9)
	No	370 (74.1)
Chronic Disease(s)	Present	257 (51.5)
	Absent	242 (48.5)
Type of Chronic Disease(s) ^a^	Hypertension	134 (52.3)
	Diabetes Mellitus	76 (29.7)
	Heart Failure	29 (11.3)
	Hyperlipidemia	8 (3.1)
	Cancer	21 (8.2)
	Asthma/COPD	36 (14.1)
	Thyroid Disease	33 (12.9)
	Other	98 (38.3)
Support Person Available	Yes	303 (60.7)
	No	196 (39.3)
Caregiving Duration (Years)	0–1	166 (33.3)
	2–5	206 (41.3)
	≥6	127 (25.5)

Note: Data are presented as frequencies (percentage). The Palliative Performance Scale (PPS) categorizes functional status as follows: Mildly Dependent (PPS ≥ 70)—able to carry out normal activity and work; Moderately Dependent (PPS 40–60)—unable to work, able to live at home with assistance; Severely Dependent (PPS ≤ 30)—unable to carry out any self-care, totally bed-bound. Support person indicates availability of additional individuals who assist with caregiving responsibilities. ^a^ = Caregivers could have more than one chronic disease; therefore, percentages may sum to more than 100%. Percentages for type of chronic disease(s) are based on the number of patients with at least one chronic disease (n = 257).

**Table 2:** Comparison of SF-36 Health Survey Scores Between Caregivers and Turkish Population Norms (n = 499).

SF-36 Domain	Caregivers	Turkish Norms	Difference	*p*-Value	Effect Size (Hedges’ g)
Physical Functioning (PF)	77.00 ± 18.93	83.8	−6.8	<0.001	−0.359 (−0.459, −0.259)
Role Physical (RP)	33.27 ± 39.33	86.3	−53.0	<0.001	−1.347 (−1.447, −1.247)
Bodily Pain (BP)	66.45 ± 27.17	82.9	−16.5	<0.001	−0.607 (−0.707, −0.507)
General Health (GH)	56.96 ± 18.69	71.6	−14.6	<0.001	−0.782 (−0.882, −0.682)
Vitality (VT)	44.03 ± 22.83	64.5	−20.5	<0.001	−0.897 (−0.997, −0.797)
Social Functioning (SF)	67.46 ± 23.06	91.0	−23.5	<0.001	−1.019 (−1.119, −0.919)
Role Emotional (RE)	47.16 ± 29.77	90.1	−42.9	<0.001	−1.442 (−1.542, −1.342)
Mental Health (MH)	51.90 ± 15.66	71.0	−19.1	<0.001	−1.218 (−1.318, −1.118)

Note: SF-36 = 36-Item Short Form Health Survey. Effect sizes are reported as Hedges’ g with 95% confidence intervals in parentheses. Effect size magnitudes were interpreted using Cohen’s criteria: small (|g| < 0.2), small-to-medium (0.2 ≤ |g| < 0.5), medium (0.5 ≤ |g| < 0.8), medium-to-large (0.8 ≤ |g| < 1.0), and large (|g| ≥ 1.0). Negative values indicate lower quality of life scores among caregivers compared with Turkish population norms. Hedges’ g provides a conservative effect size estimate and is reported with 95% CIs to facilitate precise interpretation and future meta-analyses.

**Table 3:** Distribution and Comparison of SF-36 Quality of Life Domain Scores According to Patient and Caregiver Characteristics.

Variable	Categories	n	PF	RP	BP	GH	VT	SF	RE	MH
Patient’s Age Group	65–74 years	136	79.7 ± 18.3	38.2 ± 41.2	66.8 ± 26.9	56.4 ± 18.3	41.8 ± 22.0	64.1 ± 21.8	48.5 ± 30.9	48.9 ± 15.1 ^a^
	75–84 years	189	76.9 ± 18.9	34.1 ± 38.6	68.5 ± 25.2	57.9 ± 19.1	45.9 ± 22.8	67.9 ± 25.2	47.3 ± 29.2	53.5 ± 16.1 ^b^
	≥85 years	174	75.0 ± 19.3	28.5 ± 38.3	64.0 ± 29.4	56.3 ± 18.6	43.8 ± 23.5	69.6 ± 21.4	46.0 ± 29.7	52.5 ± 15.4 ^ab^
	*p*-value		0.093	0.087	0.281	0.662	0.285	0.103	0.755	**0.027**
Patient’s Sex	Female	307	76.7 ± 18.7	33.7 ± 39.1	68.3 ± 26.5	58.5 ± 18.7	44.6 ± 22.2	68.8 ± 22.7	47.9 ± 29.5	52.6 ± 15.7
	Male	192	77.5 ± 19.3	32.6 ± 39.8	63.5 ± 28.1	54.6 ± 18.4	43.1 ± 23.8	65.4 ± 23.6	46.0 ± 30.2	50.8 ± 15.6
	*p*-value		0.627	0.749	0.059	**0.024**	0.448	0.109	0.494	0.211
Patient’s Number of Comorbidities	1	69	81.9 ± 16.7	43.5 ± 43.0	67.8 ± 23.8	56.7 ± 17.3	44.9 ± 23.1	65.8 ± 21.4	45.4 ± 31.8	50.7 ± 14.2
	2	153	77.2 ± 19.8	32.7 ± 39.8	66.8 ± 29.5	56.5 ± 20.9	44.2 ± 23.8	68.0 ± 24.8	46.6 ± 30.7	51.3 ± 16.7
	3	131	75.8 ± 19.7	31.5 ± 37.5	66.7 ± 26.5	56.5 ± 17.6	42.3 ± 22.1	66.1 ± 22.1	49.9 ± 29.1	52.1 ± 15.0
	≥4	146	75.6 ± 18.0	30.7 ± 38.3 ^b^	65.2 ± 26.9	58.0 ± 17.9	45.1 ± 22.5	68.9 ± 22.9	46.1 ± 28.6	52.9 ± 15.8
	*p*-value		0.114	0.133	0.914	0.883	0.752	0.689	0.671	0.739
Patient’s Palliative Performance Scale Score	≥70	72	80.8 ± 17.0 ^a^	50.4 ± 44.1 ^a^	75.4 ± 24.6 ^a^	60.1 ± 17.4	46.0 ± 23.0	67.2 ± 22.3	48.6 ± 30.6	51.7 ± 15.1
	60–40	322	75.1 ± 19.4 ^b^	30.1 ± 37.9 ^b^	67.0 ± 26.2 ^b^	57.1 ± 19.2	43.7 ± 23.7	68.1 ± 23.4	45.9 ± 29.0	52.6 ± 15.6
	≤30	105	80.3 ± 18.1 ^a^	31.4 ± 37.5 ^b^	58.8 ± 29.8 ^c^	54.4 ± 17.7	43.9 ± 19.8	65.6 ± 22.7	50.2 ± 31.4	49.9 ± 16.2
	*p*-value		**0.009**	**<0.001**	**<0.001**	0.134	0.735	0.617	0.397	0.314
Caregiver’s Age Group	18–64 years	424	79.4 ± 18.1	34.1 ± 39.6	67.3 ± 26.9	58.0 ± 18.8	45.2 ± 22.9	68.8 ± 23.4	46.6 ± 30.4	52.6 ± 15.7
	≥65 years	75	63.7 ± 18.2	28.3 ± 37.5	61.5 ± 28.2	51.0 ± 16.8	37.6 ± 21.8	60.0 ± 19.9	50.2 ± 25.9	47.9 ± 15.2
	*p*-value		**<0.001**	0.239	0.085	**0.003**	**0.008**	**0.002**	0.334	**0.016**
Caregiver’s Sex	Female	360	75.9 ± 18.7	31.0 ± 39.2	64.0 ± 28.2	56.8 ± 19.1	44.1 ± 23.3	67.8 ± 23.5	47.9 ± 29.5	52.5 ± 16.0
	Male	139	79.9 ± 19.2	39.2 ± 39.1	72.8 ± 23.4	57.3 ± 17.8	43.8 ± 21.7	66.6 ± 21.9	45.3 ± 30.6	50.4 ± 14.6
	*p*-value		**0.034**	**0.036**	**<0.001**	0.781	0.879	0.621	0.392	0.186
Caregiver’s Marital Status	Married	375	76.7 ± 18.5	34.9 ± 40.2	66.4 ± 26.9	56.4 ± 19.1	44.3 ± 22.5	66.5 ± 23.1	46.7 ± 30.6	51.8 ± 15.8
	Not Married	124	77.9 ± 20.2	28.2 ± 36.3	66.7 ± 28.0	58.8 ± 17.5	43.2 ± 23.9	70.3 ± 23.0	48.7 ± 27.0	52.1 ± 15.3
	*p*-value		0.524	0.100	0.915	0.210	0.636	0.119	0.519	0.873
Caregiver’s Income Level	Low	69	72.5 ± 19.9	26.5 ± 35.6	59.8 ± 28.0	50.5 ± 16.6 ^a^	38.0 ± 20.1 ^a^	64.3 ± 20.9	52.7 ± 31.0	47.4 ± 13.5 ^a^
	Middle	380	77.4 ± 18.9	34.2 ± 40.3	67.4 ± 27.1	58.0 ± 18.6 ^b^	45.5 ± 23.0 ^b^	68.6 ± 23.3	46.1 ± 29.5	53.1 ± 15.6 ^b^
	High	50	80.3 ± 17.0	35.5 ± 36.8	68.2 ± 25.4	58.3 ± 20.3 ^b^	41.4 ± 23.8 ^b^	63.0 ± 23.3	48.0 ± 29.5	49.2 ± 17.9 ^ab^
	*p*-value		0.063	0.294	0.086	**0.008**	**0.029**	0.128	0.233	**0.009**
Caregiver’s Educational Status	High school or less	379	74.9 ± 18.9	29.0 ± 38.1	65.5 ± 26.3	56.9 ± 18.6	44.3 ± 23.5	68.7 ± 22.8	47.7 ± 28.8	53.3 ± 15.4
	University or higher	120	83.5 ± 17.4	46.9 ± 40.2	69.4 ± 29.7	57.3 ± 19.0	43.1 ± 20.7	63.5 ± 23.5	45.6 ± 32.6	47.5 ± 15.7
	*p*-value		**<0.001**	**<0.001**	0.170	0.804	0.603	**0.033**	0.498	**<0.001**
Caregiver’s Employment Status	Employed	139	84.9 ± 16.3	45.5 ± 42.0	72.3 ± 26.6	61.0 ± 17.7	48.6 ± 21.5	67.8 ± 22.2	47.0 ± 32.8	52.6 ± 15.1
	Unemployed	360	74.0 ± 19.0	28.5 ± 37.3	64.2 ± 27.1	55.4 ± 18.9	42.3 ± 23.1	67.3 ± 23.4	47.2 ± 28.6	51.6 ± 15.9
	*p*-value		**<0.001**	**<0.001**	**0.003**	**0.003**	**0.006**	0.835	0.941	0.555
Does caregiver Receive Caregiving Salary?	No	394	76.2 ± 19.1	32.7 ± 39.7	67.3 ± 26.2	57.6 ± 19.1	44.6 ± 24.3	69.5 ± 23.4	48.3 ± 28.8	53.4 ± 15.9
	Yes	105	80.2 ± 17.9	35.2 ± 37.9	63.2 ± 30.4	54.5 ± 16.8	41.9 ± 16.3	59.6 ± 19.9	42.9 ± 32.9	46.3 ± 13.3
	*p*-value		**0.045**	0.554	0.209	0.106	0.182	**<0.001**	0.124	**<0.001**
Does caregiver have Other Care Recipients?	Yes	129	73.6 ± 18.7	25.8 ± 36.7	63.5 ± 28.3	54.9 ± 19.2	42.3 ± 24.4	67.0 ± 25.0	45.5 ± 29.7	52.9 ± 17.0
	No	370	78.2 ± 18.9	35.9 ± 39.9	67.5 ± 26.7	57.7 ± 18.5	44.6 ± 22.3	67.6 ± 22.4	47.8 ± 29.8	51.6 ± 15.2
	*p*-value		**0.016**	**0.012**	0.156	0.142	0.315	0.774	0.456	0.412
Does caregiver have Comorbidities?	Yes	257	70.8 ± 17.6	32.0 ± 39.5	62.9 ± 27.0	53.1 ± 18.8	40.9 ± 22.4	64.0 ± 22.2	46.0 ± 29.2	50.4 ± 15.4
	No	242	83.6 ± 18.1	34.6 ± 39.2	70.3 ± 26.9	61.1 ± 17.7	47.3 ± 22.9	71.2 ± 23.5	48.4 ± 30.4	53.5 ± 15.8
	*p*-value		**<0.001**	0.460	**0.002**	**<0.001**	**0.002**	**<0.001**	0.388	**0.029**
Does caregiver have a Support Person Available?	Yes	303	79.4 ± 18.3	34.9 ± 39.6	66.7 ± 27.8	58.4 ± 18.4	44.7 ± 22.7	70.0 ± 22.4	48.1 ± 31.3	52.9 ± 15.7
	No	196	73.3 ± 19.3	30.7 ± 38.9	66.1 ± 26.3	54.7 ± 19.0	43.0 ± 23.1	63.5 ± 23.6	45.8 ± 27.2	50.4 ± 15.5
	*p*-value		**<0.001**	0.249	0.797	**0.031**	0.412	**0.002**	0.394	0.078
Caregiver’s Caregiving Duration (Years)	0–1	166	81.2 ± 17.8 ^a^	39.2 ± 41.5	69.3 ± 28.2	58.7 ± 19.2	46.6 ± 24.2	70.2 ± 23.1	45.8 ± 31.9	51.5 ± 16.5
	2–5	206	77.1 ± 18.6 ^a^	30.8 ± 37.8	65.2 ± 26.5	56.3 ± 19.0	42.6 ± 22.9	67.7 ± 22.5	49.5 ± 28.7	52.1 ± 15.3
	≥6	127	71.3 ± 19.6 ^b^	29.5 ± 38.2	64.7 ± 26.8	55.8 ± 17.5	43.1 ± 20.6	63.6 ± 23.5	45.1 ± 28.7	52.1 ± 15.1
	*p*-value		**<0.001**	0.059	0.245	0.345	0.211	0.052	0.329	0.905

Note: Data are presented as the mean ± standard deviation. *p*-values were derived from an independent samples *t*-test for comparisons between two groups and one-way ANOVA for comparisons across three or more groups. For variables with three or more categories, post hoc analyses were conducted using Tukey’s HSD test. Statistical significance was set at *p* < 0.05; significant *p*-values are indicated in bold. Superscript letters (a, b, c) indicate statistically significant differences between subgroups based on post hoc Tukey HSD tests (*p* < 0.05). Groups that share the same superscript letter do not differ significantly from each other, whereas groups with different superscript letters have significantly different mean scores. For the Role Physical (RP) domain, which deviated from normality, comparisons were performed using non-parametric tests (Mann–Whitney U or Kruskal–Wallis); mean ± SD values are presented for descriptive purposes only. Abbreviations: SF-36: 36-Item Short Form Health Survey; PF: Physical Functioning; RP: Role Physical; BP: Bodily Pain; GH: General Health; VT: Vitality; SF: Social Functioning; RE: Role Emotional; MH: Mental Health; SD: Standard Deviation; ANOVA: Analysis of Variance; HSD: Honest Significant Difference.

**Table 4:** Distribution of SF-36 Physical Component Summary (PCS) and Mental Component Summary (MCS) Scores According to Patient and Caregiver Characteristics (n = 499).

Variable	n	PCS Score	*p*-Value	MCS Score	*p*-Value
Patient Age Group					
65–74 years	136	60.3 ± 17.9	0.090	50.8 ± 16.7	0.328
75–84 years	189	59.4 ± 19.1		53.6 ± 17.9	
≥85 years	174	55.9 ± 19.3		53.0 ± 16.6	
Patient Sex			0.201		0.168
Female	307	59.3 ± 18.5		53.5 ± 17.0	
Male	192	57.1 ± 19.6		51.3 ± 17.3	
Patient Number of Comorbidities			0.276		0.940
1	69	62.5 ± 17.6		51.7 ± 16.1	
2	153	58.3 ± 20.4		52.5 ± 18.7	
3	131	57.6 ± 18.2		52.6 ± 16.3	
≥4	146	57.4 ± 18.5		53.3 ± 16.7	
Patient Palliative Performance Scale (PPS)			**<0.001**		0.922
Mildly Dependent (≥70)	72	66.7 ± 18.7 ^a^		53.4 ± 18.0	
Moderately Dependent (40–60)	322	57.3 ± 18.9 ^b^		52.6 ± 17.3	
Severely Dependent (≤30)	105	56.2 ± 17.9 ^b^		52.4 ± 16.1	
Caregiver Age Group			**<0.001**		**0.041**
18–64 years	424	59.7 ± 18.9		53.3 ± 17.4	
≥65 years	75	51.1 ± 17.3		48.9 ± 15.1	
Caregiver Sex			**0.004**		0.373
Female	360	56.9 ± 19.2		53.1 ± 17.5	
Male	139	62.3 ± 17.6		51.5 ± 16.2	
Caregiver Marital Status			0.728		0.494
Married	375	58.6 ± 19.2		52.3 ± 17.4	
Not Married	124	57.9 ± 18.2		53.6 ± 16.3	
Caregiver Income Level			**0.013**		0.298
Low	69	52.3 ± 17.7 ^a^		50.6 ± 15.1	
Middle	380	59.3 ± 19.0 ^b^		53.3 ± 17.4	
High	50	60.6 ± 18.8 ^b^		50.4 ± 17.7	
Caregiver Education Level			**<0.001**		**0.046**
High School or Less	379	56.6 ± 18.6		53.5 ± 17.0	
University or Higher	120	64.3 ± 18.8		49.9 ± 17.2	
Caregiver Employment Status			**<0.001**		0.275
Employed	139	65.9 ± 18.1		54.0 ± 17.4	
Not Employed	360	55.5 ± 18.4		52.1 ± 17.0	
Receipt of Caregiving Payment			0.937		**<0.001**
No Payment	394	58.5 ± 19.5		54.0 ± 17.6	
Receives Payment	105	58.3 ± 16.8		47.7 ± 14.0	
Additional Care Recipients			**0.005**		0.570
Yes	129	54.4 ± 18.4		51.9 ± 18.3	
No	370	59.8 ± 18.9		52.9 ± 16.7	
Caregiver’s Chronic Illness			**<0.001**		**0.002**
Present	257	54.7 ± 19.0		50.3 ± 16.7	
Absent	242	62.4 ± 18.1		55.1 ± 17.3	
Support Person Available			**0.035**		**0.037**
Yes	303	59.9 ± 18.9		53.9 ± 17.0	
No	196	56.2 ± 18.8		50.7 ± 17.1	
Caregiving Duration (years)			**0.006**		0.431
0–1 years	166	62.1 ± 19.9 ^a^		53.5 ± 18.4	
2–5 years	206	57.4 ± 18.3 ^ab^		53.0 ± 16.4	
≥6 years	127	55.3 ± 17.9 ^b^		51.0 ± 16.5	

Note: PCS = Physical Component Summary (mean of Physical Functioning, Role Physical, Bodily Pain, and General Health domains); MCS = Mental Component Summary (mean of Vitality, Social Functioning, Role Emotional, and Mental Health domains). Scores range from 0 to 100; higher scores indicate better health-related quality of life. Turkish population reference values: PCS ≈ 81.2, MCS ≈ 79.2 (derived from Demiral et al., 2006 [28]). Data are presented as mean ± standard deviation. *p*-values were derived from independent-samples *t*-tests (two groups) or one-way ANOVA (three or more groups). Bold *p*-values indicate statistical significance at *p* < 0.05. Superscript letters (a, b) denote post hoc Tukey HSD groupings (*p* < 0.05); groups sharing the same superscript letter are not significantly different. Abbreviations: PCS = Physical Component Summary; MCS = Mental Component Summary; PPS = Palliative Performance Scale; SD = Standard Deviation; ANOVA = Analysis of Variance; HSD = Honest Significant Difference.

**Table 5:** Multivariate Linear Regression Analysis of Factors Associated with Caregivers’ Physical (PCS) and Mental (MCS) Component Summary Scores (n = 499).

Variable	PCS			MCS		
	B (95% CI)	β	*p*-Value	B (95% CI)	β	*p*-Value
Patient PPS Level (ref: Mildly Dependent, PPS ≥ 70)						
Moderately Dependent (PPS 40–60)	−8.56 (−13.14, −3.98)	−0.217	**<0.001**	−2.07 (−6.45, 2.32)	−0.058	0.355
Severely Dependent (PPS ≤ 30)	−12.05 (−17.44, −6.65)	−0.260	**<0.001**	−0.68 (−5.85, 4.48)	−0.016	0.795
Age ≥ 65 years (ref: 18–64 years)	−4.72 (−9.32, −0.12)	−0.089	**0.044**	−2.86 (−7.27, 1.54)	−0.060	0.202
Male Sex (ref: Female)	3.25 (−0.39, 6.89)	0.077	0.080	−1.54 (−5.03, 1.95)	−0.040	0.387
Income Level (ref: Low)						
Middle Income	2.81 (−1.92, 7.54)	0.063	0.243	1.23 (−3.29, 5.76)	0.031	0.593
High Income	2.89 (−3.95, 9.73)	0.046	0.407	1.42 (−5.12, 7.97)	0.025	0.669
University Education or Higher (ref: High School or Less)	3.45 (−0.85, 7.74)	0.078	0.115	−4.31 (−8.42, −0.20)	−0.108	**0.040**
Unemployed (ref: Employed)	−4.98 (−8.95, −1.01)	−0.118	**0.014**	−1.84 (−5.64, 1.96)	−0.048	0.342
Receives Caregiving Payment (ref: No Payment)	−1.89 (−6.02, 2.23)	−0.041	0.368	−5.76 (−9.71, −1.81)	−0.137	**0.004**
Additional Care Recipients Present (ref: No)	−4.10 (−7.71, −0.49)	−0.095	**0.026**	−0.81 (−4.27, 2.65)	−0.021	0.646
Chronic Illness Present (ref: Absent)	−6.40 (−9.58, −3.22)	−0.169	**<0.001**	−4.71 (−7.75, −1.67)	−0.138	**0.002**
Support Person Available (ref: No)	1.58 (−1.74, 4.90)	0.041	0.351	3.37 (0.19, 6.55)	0.096	**0.038**
Caregiving Duration (ref: 0–1 years)						
2–5 years	−3.23 (−6.87, 0.40)	−0.084	0.081	−0.30 (−3.78, 3.18)	−0.009	0.865
≥6 years	−3.73 (−7.95, 0.49)	−0.086	0.083	−0.79 (−4.83, 3.24)	−0.020	0.699

PCS Model: R^2^ = 0.169, Adjusted R^2^ = 0.145, F (14, 484) = 7.041, *p* < 0.001. MCS Model: R^2^ = 0.071, Adjusted R^2^ = 0.044, F (14, 484) = 2.631, *p* = 0.001. Multicollinearity: All Variance Inflation Factor (VIF) values < 5.0 (range: 1.34–4.94); no multicollinearity concern. Note: PCS = Physical Component Summary (mean of Physical Functioning, Role Physical, Bodily Pain, and General Health domains); MCS = Mental Component Summary (mean of Vitality, Social Functioning, Role Emotional, and Mental Health domains). Scores range from 0 to 100; higher scores indicate better health-related quality of life. B = unstandardized regression coefficient; β = standardized regression coefficient; 95% CI = 95% confidence interval; PPS = Palliative Performance Scale. Bold *p*-values indicate statistical significance at *p* < 0.05.

## Data Availability

The data presented in this study are only available from the corresponding author upon request due to privacy and ethical reasons.

## References

[1] de Oliveira Tavares M.L., Pimenta A.M., Garcia-Vivar C., Beinner M.A., Montenegro L.C. (2025). Determinants of quality of life decrease in family caregivers of care-dependent patients: A longitudinal study. Qual. Life Res..

[2] Bernal-Alonso A., Rodriguez-Blazquez C., Fernandez-Carro C., Ayala A., Calderon-Larranaga A., Forjaz M.J. (2025). Quality of life of informal caregivers 50 years old and older in Spain by household living status: A descriptive study. Sci. Rep..

[3] Possin K.L., Dulaney S., Sideman A.B., Wood A.J., Allen I.E., Bonasera S.J., Merrilees J.J., Lee K., Chiong W., Braley T.L. (2025). Long-term effects of collaborative dementia care on quality of life and caregiver well-being. Alzheimers Dement..

[4] Barben J., Billa O., Collot J., Collot T., Manckoundia P., Bengrine-Lefevre L., Dabakuyo-Yonli T.S., Quipourt V. (2023). Quality of life and perceived burden of the primary caregiver of patients aged 70 and over with cancer 5 years after initial treatment. Support. Care Cancer.

[5] Zhang Y., Li J., Zhang Y., Chen C., Guan C., Zhou L., Zhang S., Chen X., Hu X. (2024). Mediating effect of social support between caregiver burden and quality of life among family caregivers of cancer patients in palliative care units. Eur. J. Oncol. Nurs..

[6] Ullrich A., Wortberg E., Bokemeyer C., Oechsle K. (2025). Psychological distress, quality of life, needs, and resources among informal caregivers in specialist palliative home care. Support. Care Cancer.

[7] Haroen H., Maulana S., Harun H., Mirwanti R., Sari C.W.M., Platini H., Arovah N.I., Padila P., Amirah S., Pardosi J.F. (2025). The benefits of early palliative care on psychological well-being, functional status, and health-related quality of life among cancer patients and their caregivers: A systematic review and meta-analysis. BMC Palliat. Care.

[8] Alaei A., Babaei S., Farzi S., Hadian Z. (2024). Effect of a supportive-educational program, based on COPE model, on quality of life and caregiver burden of family caregivers of heart failure patients: A randomized clinical trial study. BMC Nurs..

[9] Sambasivam R., Liu J., Vaingankar J.A., Ong H.L., Tan M.E., Fauziana R., Picco L., Chong S.A., Subramaniam M. (2019). The hidden patient: Chronic physical morbidity, psychological distress, and quality of life in caregivers of older adults. Psychogeriatrics.

[10] Rostami M., Abbasi M., Soleimani M., Moghaddam Z.K., Zeraatchi A. (2023). Quality of life among family caregivers of cancer patients: An investigation of SF-36 domains. BMC Psychol..

[11] Lillekroken D., Bjorge H., Halvorsrud L., Lidal I.B. (2025). Assessing quality of life—A scoping review of studies presenting quality of life instruments for informal caregivers of persons with dementia. BMC Geriatr..

[12] Marinacci L.X., Sterling M.R., Zheng Z., Wadhera R.K. (2025). Health-Related Quality of Life of Family Caregivers in the United States, 2021–2022: A National Cross-Sectional Analysis. J. Gen. Intern. Med..

[13] Tay L.X., Ong S.C., Ong H.M., Teh E.E., Ch’ng A.S.H., Tiong I.K., Razali R.M., Parumasivam T. (2025). Assessing the health-related quality of life in informal caregivers of Alzheimer’s Disease: Evidence from Malaysia. BMC Palliat. Care.

[14] Garcia-Torres F., Jablonski M.J., Gomez-Solis A., Moriana J.A., Jaen-Moreno M.J., Moreno-Diaz M.J., Aranda E. (2022). Anxiety, depression and quality of life: A longitudinal study involving cancer patient-caregiver dyads. Health Psychol. Rep..

[15] Kim Y.M., Lee J.E. (2023). Dyadic Effects of Psychological Health on Quality of Life in Patients with Colorectal Cancer and Caregivers: A Systematic Review and Meta-Analysis. Semin. Oncol. Nurs..

[16] Cubukcu M. (2018). Evaluation of quality of life in caregivers who are providing home care to cancer patients. Support. Care Cancer.

[17] Ugur H.G., Erci B. (2019). The Effect of Home Care for Stroke Patients and Education of Caregivers on the Caregiver Burden and Quality of Life. Acta Clin. Croat..

[18] Liu J., Zhang Y., Guan T., Wang X., Ma C., Northouse L., Song L. (2025). Quality of life and appraisal factors of patients with advanced cancer and their family caregivers. Support. Care Cancer.

[19] Yang Y., Deng Q., Liu L., Chen Y. (2025). The Impact of Caregiving Intensity and Financial Burden on the Health-Related Quality of Life Among Informal Caregivers for Patients with Advanced Lung Cancer: A Multicenter Cross-Sectional Study. J. Nurs. Manag..

[20] Sadeghi A., Yousofvand V., Falahan S.N., Bonyad S.A., Alafchi B. (2025). Effects of virtual counseling on the care burden and quality of life of family caregivers for leukemia patients: A randomized controlled trial study. BMC Nurs..

[21] Seok Y., Lee M.K. (2025). Health-related quality of life predictors for patients with stroke: A prospective longitudinal study of matched pairs of patients with stroke and family caregivers. J. Clin. Nurs..

[22] Anderson F., Downing G.M., Hill J., Casorso L., Lerch N. (1996). Palliative performance scale (PPS): A new tool. J. Palliat. Care.

[23] Virik K., Glare P. (2002). Validation of the palliative performance scale for inpatients admitted to a palliative care unit in Sydney, Australia. J. Pain Symptom Manag..

[24] Oğuz G., Şenel G., Koçak N., Karaca Ş (2021). The Turkish Validity and Reliability Study of Palliative Performance Scale. Asia Pac. J. Oncol. Nurs..

[25] Baik D., Russell D., Jordan L., Dooley F., Bowles K.H., Masterson Creber R.M. (2018). Using the Palliative Performance Scale to Estimate Survival for Patients at the End of Life: A Systematic Review of the Literature. J. Palliat. Med..

[26] Ware J.E., Sherbourne C.D. (1992). The MOS 36-item short-form health survey (SF-36). I. Conceptual framework and item selection. Med. Care.

[27] Koçyiğit H., Aydemir O., Fişek G., Olmez N., Memiş A. (1999). Kısa Form-36 (SF-36)’nın Türkçe Versiyonunun Güvenilirliği ve Geçerliliği. [Reliability and Validity of the Turkish Version of Short Form-36 (SF-36)]. İlaç Tedavi Derg..

[28] Demiral Y., Ergor G., Unal B., Semin S., Akvardar Y., Kivircik B., Alptekin K. (2006). Normative data and discriminative properties of short form 36 (SF-36) in Turkish urban population. BMC Public Health.

[29] Lakens D. (2013). Calculating and reporting effect sizes to facilitate cumulative science: A practical primer for t-tests and ANOVAs. Front. Psychol..

[30] Franchini L., Ercolani G., Ostan R., Raccichini M., Samolsky-Dekel A., Malerba M.B., Melis A., Varani S., Pannuti R. (2020). Caregivers in home palliative care: Gender, psychological aspects, and patient’s functional status as main predictors for their quality of life. Support. Care Cancer.

[31] Alnaeem M.M., Al Qadire M., Nashwan A.J. (2025). Unmet needs, burden, and quality of life among family caregivers of patients with advanced hematological malignancy. Psychol. Health Med..

[32] Mirhosseini S., Imani Parsa F., Moghadam-Roshtkhar H., Basirinezhad M.H., Ameri M., Ebrahimi H. (2025). Support based on psychoeducation intervention to address quality of life and care burden among caregivers of patients with cancer: A randomized controlled trial. Front. Psychol..

[33] Kim J., Lee S. (2025). The effect of unmet needs on the health-related Quality of life of family caregivers of cancer patients in South Korea. PLoS ONE.

[34] Wang S., Dong J., Wen L., Tang W., Zhang X., Fu J., Zhu J., Wang Y., Zhang H., Lyaruu L.I. (2025). Relationship between quality of life of patients with severe mental illnesses and family burden of disease: The mediating effect of caregivers’ social support. BMC Public Health.

[35] Baudry A.S., Delpuech M., Charton E., Peugniez C., Hivert B., Carnot A., Ceban T., Dominguez S., Lemaire A., Aelbrecht-Meurisse C. (2025). Is intrapersonal emotional competence a personal resource for the quality of life of informal caregivers of cancer patients unlike interpersonal emotional competence?. Qual. Life Res..

[36] Cui P., Ai J., Chen X., Cheng C., Shi J., Li S., Yang M., Chen C., Hu H. (2025). Dyadic effects of perceived burden and psychological distress on quality of life among Chinese advanced cancer patients and their caregivers. Sci. Rep..

[37] Sawatzky R., Schick-Makaroff K., Ratner P.A., Kwon J.Y., Whitehurst D.G.T., Ohlen J., Maybee A., Stajduhar K., Zetes-Zanatta L., Cohen S.R. (2025). Did a digital quality of life (QOL) assessment and practice support system in home health care improve the QOL of older adults living with life-limiting conditions and of their family caregivers? A mixed-methods pragmatic randomized controlled trial. PLoS ONE.

